# Large-amplitude internal waves benefit corals during thermal stress

**DOI:** 10.1098/rspb.2014.0650

**Published:** 2015-01-22

**Authors:** M. Wall, L. Putchim, G. M. Schmidt, C. Jantzen, S. Khokiattiwong, C. Richter

**Affiliations:** 1 Alfred Wegener Institute, Helmholtz Center for Polar and Marine Research, Am Alten Hafen 26, 27568 Bremerhaven, Germany; 2 GEOMAR, Helmholtz Center for Ocean Research, Marine Geosystems, Wischhofstraße 1-3, 24148 Kiel, Germany; 3 Phuket Marine Biological Center, 51 Sakdidet Road, 83000 Phuket, Thailand

**Keywords:** coral bleaching, large-amplitude internal waves, solitons, cooling, global warming

## Abstract

Tropical scleractinian corals are particularly vulnerable to global warming as elevated sea surface temperatures (SSTs) disrupt the delicate balance between the coral host and their algal endosymbionts, leading to symbiont expulsion, mass bleaching and mortality. While satellite sensing of SST has proved a reliable predictor of coral bleaching at the regional scale, there are large deviations in bleaching severity and mortality on the local scale that are poorly understood. Here, we show that internal waves play a major role in explaining local coral bleaching and mortality patterns in the Andaman Sea. Despite a severe region-wide SST anomaly in May 2010, frequent upslope intrusions of cold sub-pycnocline waters due to breaking large-amplitude internal waves (LAIW) mitigated coral bleaching and mortality in shallow waters. In LAIW-sheltered waters, by contrast, bleaching-susceptible species suffered severe bleaching and total mortality. These findings suggest that LAIW benefit coral reefs during thermal stress and provide local refugia for bleaching-susceptible corals. LAIW are ubiquitous in tropical stratified waters and their swash zones may thus be important conservation areas for the maintenance of coral diversity in a warming climate. Taking LAIW into account can significantly improve coral bleaching predictions and provide a valuable tool for coral reef conservation and management.

## 1. Introduction

Global warming and ocean acidification are recognized as the major threats to coral reefs [1,2]. The thermal optimum for most scleractinian corals is very close to their upper thermal temperature limit, and therefore moderate increases in sea surface temperatures (SSTs) of 1–2°C can become stressful to corals [3,4]. Such stressful conditions are known to disrupt the photosymbioses between corals and the unicellular algae *Symbiodinium,* thus causing symbiont loss, coral bleaching and mortality [3–5]. A series of global mass bleaching events has led to a marked decline in coral cover and species diversity over recent decades [2,6], and concerns over the projected increase in frequency and intensity of bleaching events with the eventual demise of coral reefs [7] has fostered the search for natural refugia [8,9].

Reef refugia maintain higher coral cover and species diversity, and are target areas for reef conservation. Both extrinsic and intrinsic factors may contribute to coral reef resistance to thermal stress [8,10]. Several studies in various environmental settings have confirmed that extrinsic environmental factors such as mixing and advection of cooler water (e.g. in upwelling regions or offshore reefs) can alleviate heating and provide refuge from bleaching [9,11–13]. More recently, high-frequency step changes in temperature were observed in Indian Ocean and Andaman Sea coral reefs [14–16], most probably due to breaking large-amplitude internal waves (LAIW) [17,18]. LAIW are particularly strong during periods of maximum thermal stratification and SSTs [15], ubiquitous in the world ocean [19], and observed to reach into many coral reef environments [15,16,20–28]. However, their potential role in mitigating thermal stress has not yet been investigated.

LAIW are generated when strong tidal flows interact with topographic features and travel along the density gradient in the water column. In the Andaman Sea, the Andaman–Nicobar Island arc and shallow Dreadnought Bank generate internal waves with extraordinary large amplitudes of up to 80 m that travel eastwards with speeds of approximately 2 m s^−1^ [18,29]. When they approach the Thai continental slope and shelf, they transform into secondary wave trains [30]. These waves of elevation with trapped recirculating cores may propagate for considerable lengths across the shelf bottom [31] and carry parcels of cold subpycnoline water into shallower coral reef areas [15,16]. The temperature drops are sudden (within minutes), large (up to 10°C), short (15–30 min duration), intermittent (several per cycle) and confined to the sea bed, rarely extending to the sea surface, so that they are largely invisible to remote temperature sensing by satellites [14,32].

A monsoonal climate dominates the Andaman Sea. April/May marks the transition from northeast (NE) to southwest (SW) monsoon with peak annual temperatures [33]. During the dry NE monsoon season, when the pycnocline shoals, LAIW are strongest (January through March). During the SW monsoon season, by contrast, the pycnocline is generally deeper. Southwesterly winds pile up surface water and depress the pycnocline so that fewer LAIW propagate upslope and reach into shallow reef areas [15,16]. Around July/August, the SW monsoon reaches its full intensity with advection and turbulent mixing, increasing resuspension of sediments in shallow water [16]. Both LAIW and the SW monsoon act from the same westerly direction so that west island sides are exposed to both internal and surface waves, albeit at different times, whereas eastern sides remain sheltered. Weak LAIW and monsoon mixing may overlap during transition seasons.

Although the Andaman Sea has experienced major coral bleaching events in 1991, 1995 and 2003, the 2010 mass bleaching event was the most severe on record. It caused high loss of live coral cover, but showed pronounced local differences in bleaching extent and subsequent mortality, which may be attributed in part to local differences in coral community composition (intrinsic factor) with more or less bleaching-susceptible species [34,35]. Part of the variability may also be speculated to be due to internal waves [34,35], but a test of this hypothesis is lacking. Thus, the 2010 severe bleaching event provides an excellent opportunity to test the underlying hypothesis: can LAIW benefit reefs during mass bleaching?

**Figure 1.**
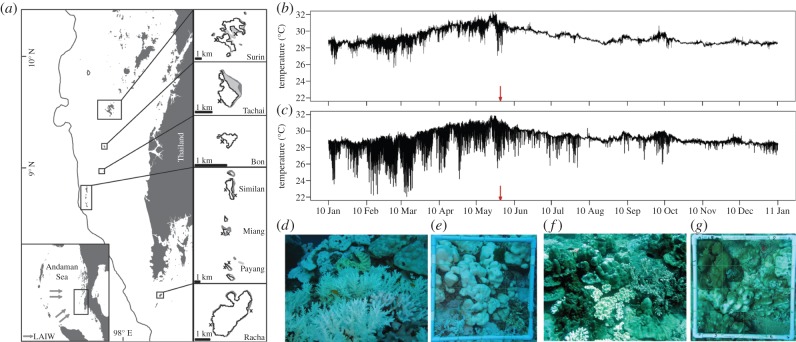
Study sites, temperature and BR at exposed and sheltered island sides in the Andaman Sea, Thailand. (*a*) Study sites on the Thai continental shelf beyond the breaking zone of LAIW near the 200 m isobath (line). Lower left inset shows the Andaman Sea with the direction of LAIW propagation (and monsoon winds). Right insets show close-ups of the islands with locations of the of study sites on opposing (exposed and sheltered) sides of the islands (sources of maps: mainland, Wessel & Smith [36]; bathymetry, Smith & Sandwell [37]; study islands: UNEP Coral Millennium Project). (*b*–*g*) Temperature and BR observed on the (*b*,*d*,*e*) sheltered and (*c*,*f*,*g*) exposed island sides of Miang. (*b*,*c*) The red arrow in the temperature graphs marks the time of bleaching monitoring. (*d*–*g*) Images display the observed difference in BR between (*d*,*e*) sheltered and (*f*,*g*) exposed island sites.

We took advantage of the natural setting of the Thai continental shelf (i.e. coral-fringed islands with differential exposure to LAIW [16]; figure 1*a*) and we took into account species-specific differences in coral susceptibility to heat stress. We hypothesize that differences in bleaching response (BR) are inversely related to LAIW exposure and a function of differences in community composition. If LAIW are able to reduce heat stress and mitigate coral bleaching, this would have important implications for reef health in the future and should be considered in coral reef conservation and management.

## 2. Material and methods

### (a) Study sites

Seven islands were chosen for this study located on the continental shelf west of Thailand in the Andaman Sea. From north to south, the islands were Surin, Tachai, Bon, Similan, Miang, Payang and Racha (figure 1*a*). Twelve sites were selected; seven sites facing west (W) were exposed to LAIW and SW monsoon impact (Racha, Payang, Miang, Similan, Bon, Tachai and Surin), whereas five other sites were located on the LAIW and SW monsoon sheltered east (E) island sides (Racha, Payang, Miang, Similan and Surin).

### (b) Environmental background

*In situ* temperature was recorded with Onset HOBO temperature loggers (Tidbits; resolution: ±0.2°C). They were deployed in 15 m water depth at Racha W, Miang E, Miang W, Bon W, Tachai W and Surin W, logging at 3 min intervals for the entire year 2010 [16]*.* At Racha E, loggers were deployed at 20 and 10 m with a logging interval of 20 min and recorded the temperature from March 2010 until the end of July 2010. For Surin E, a temperature record was available from 15 m water depth ranging from March 2010 to December 2010 with a logging interval of 20 min (data courtesy of the Phuket Marine Biological Center from their Andaman Sea Monitoring Programme). Temperature data are unavailable for Payang and Similan, but previous work has shown only marginal temperature differences between similarly exposed sides of the Similan Islands (figure 1*a*), so that the temperature records available for the east and west sides of the central Similan Island Miang can be taken as representative [15].

### (c) Bleaching survey

At each of the 12 sites, photoframe (50 × 50 cm) images were taken at the study depth of 15 m with 31–70 quadrats per site during the May 2010 (high temperature anomaly) and 27–80 quadrats per site during the December 2010 sampling (recovery phase). The sampling procedure involved placing the frames randomly into the reef following the 15 m isobath over a distance of 25–50 m and taking photographs perpendicular to the substrate with the frame in the centre of the image. Photos were taken with Canon Powershot G12 cameras with underwater housing (resolution: minimum 3648 × 2736 pixels per image).

### (d) Data analyses

#### (i) Temperature analyses

We used both satellite-derived degree heating weeks (DHW_s_) from the National Oceanographic and Atmospheric Administration (NOAA) and *in situ* field data to calculate degree heating weeks (DHW_f_) according to NOAA (see the electronic supplementary material for more details). We used regression models to compare the ability of both satellite and *in situ* records to predict bleaching in the Andaman Sea.

LAIW cooling intensities were quantified for each site by calculating cumulative degree-day cooling values according to Leichter & Genovese [20] (see the electronic supplementary material for more detail).

#### (ii) Photoframe analyses

Photoframe images were processed with the coral point count method (CPCe; cf. [38]) to determine the percentage live and dead coral cover, coral community composition and bleaching status of the corals. A uniform grid of 15 × 15 points was superimposed on each frame and the presence of live or dead corals beneath each point recorded. The bleaching status of the coral at each point was assessed on an ordinal scale ranging from ‘healthy’ (with usual pigmentation) to ‘pale’ (reduced pigmentation), ‘bleached’ (completely white tissue), ‘recently dead’ (where the bare white skeleton was visible and already started to be overgrown by fresh green algae) and ‘dead’ (non-white carbonate structure that is still recognizable as former coral colony; see the electronic supplementary material, figure S1). With the exception of the last category, we distinguished between the following coral groups: *Porites* spp. branching, *Porites* spp. massive, Pocilloporidae (*Pocillopora* spp. and *Stylophora* spp.), *Acropora* spp., *Diploastrea heliopora* and ‘other’ (all remaining taxa). The most dominant genera were selected (note: *Diploastrea* is a genus that only consists of one species) and all other genera were grouped together due to their relatively low abundances. These recorded coral groups were used to estimate differences in bleaching susceptibility for the coral communities at the different sites.

#### (iii) Site-specific community bleaching susceptibility index

A community bleaching susceptibility index (CBSI) was calculated by ranking the six recorded coral groups (see above) according to their reported susceptibility [34,39] into three bleaching susceptibility groups (0–2): very low susceptibility (*Diploastrea heliopora* (*s*_1_)), moderate susceptibility (*Porites* spp. massive (*s*_2_) and other (*s*_3_)) and high susceptibility (*Acropora* spp. (*s*_4_), *Porites* spp. branching (*s*_5_) and Pocilloporidae. (*s*_6_)). This index was calculated as
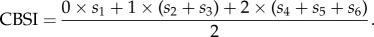
The coral group occurrences (i.e. percentage coral cover excluding ‘dead’ coral category) were multiplied by their susceptibility score and the resulting sum was normalized to a scale from 0 to 100 by dividing it by 2.

#### (iv) Site-specific bleaching response

The BR was quantified for each site [40]. This evaluation is based on the photoframe data and calculated as
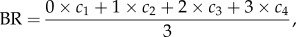
with the status-categories *c*_1_ = healthy, *c*_2_ = pale, *c*_3_ = bleached and *c*_4_ = recently dead (excluding ‘dead’ coral as mortalities cannot be derived from the particular bleaching event), all given as percentage cover for each site. The percentage coral of each category (*c*_1_–*c*_4_) was multiplied by a score (0–3) to weigh the different categories according to their bleaching intensity: no weight (0) for healthy (not bleached) and highest weight (3) for recently dead corals (i.e. mortality as a consequence of heat stress). The resulting sum was normalized to a scale from 0 to 100 by dividing it by 3. As some corals still showed signs of bleaching during the December survey, the same categories as in May were applied (‘healthy’, ‘pale’, ‘bleached’, ‘recently dead’ and ‘dead’; see electronic supplementary material, figure S1b1–2; ‘recently dead’ are corals with white tissue and/or bare skeleton overgrown by algae). This allowed calculating BR values for December that quantified the progress in recovery of the remaining corals.

For both indices, the multipliers were chosen following McClanahan *et al*. [40]. Different multipliers affect the absolute but not the relative values, and have a negligible effect on the statistical results.

In the December surveys, it was not possible to assess the time of mortality (i.e. to differentiate between the corals that had died in May and those that had already died before the bleaching event). Hence, to determine post-bleaching mortality, the ‘dead’ corals from the May survey were subtracted from the total mortality (‘dead’ and ‘recently dead’) in December.

#### (v) Bleaching response as a function of extrinsic and intrinsic variables

The ability of different extrinsic and intrinsic factors (i.e. DHW and CBSI, respectively) to predict the observed site-specific BR was tested using simple and mixed multiple linear regression models, labelled (a) to (f) [41]. They were calculated across all study sites using single predictors or a combination of predictors. Significant regression models were tested for error normal distribution (Anderson–Darling test for regression model: (b) *p* = 0.523, (c) *p* = 0.739, (e) *p* = 0.971, (f) *p* = 0.864) and homoscedasticity of errors (Breusch–Pagan test for regression model: (b) *p* = 0.153, (c) *p* = 0.788, (e) *p* = 0.223, (f) *p* = 0.209). Independence of errors (Durbin–Watson statistic for regression model: (b) = 0.300, *p* = 0.523; (c) = 0.233, *p* = 0.739; (e) = 1.88, *p* = 0.526; (f) = 2.04, *p* = 0.634; all no autocorrelation) and correlation between predictors (Spearman correlation calculated for regression model: (e) *ρ* = 0.17, *p* = 0.589, (f) *ρ* = 0.20, *p* = 0.552; both no correlation) were tested for all significant models and the multiple linear models, respectively. The best-fit multiple model (regression model (e)) was further tested for influential cases using outlier tests and Cook's statistics. Tachai W was identified for both tests as a potential influential case (outlier test: Tachai W rstudent = 2.05, *p* = 0.892; Cook's statistic: Tachai W has the highest value of 0.22, but is below the critical value of 0.44). Tachai W was colonized by a high percentage of corallimorpharian compared with the other sites (see the electronic supplementary material, table S1) that could have been additionally stressful to the corals (see Results and discussion). Thus, a new model (model (f)) was fitted with the omission of this point. The software package R (version 3.0.1) was used for all statistical analyses.

## 3. Results and discussion

**Figure 2.**
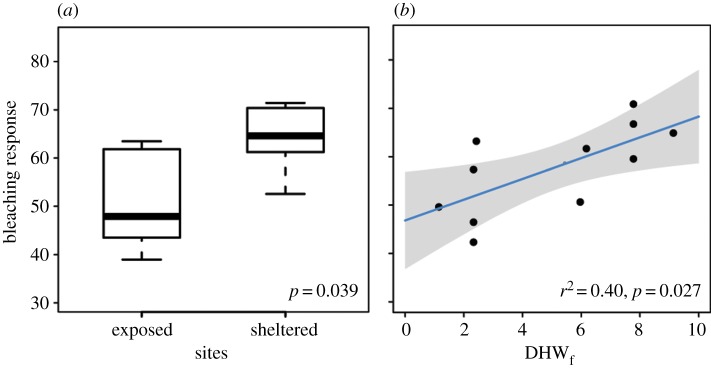
Coral reef community BR to the thermal stress in May 2010. (*a*) Boxplots display the BR (BR_M_) observed for exposed and sheltered island sites (two-tailed *t*-test, *p* = 0.039; central boxes show median and 25th and 75th percentiles, and whiskers the min. and max. range). (*b*) BR_M_ plotted as a function of heat stress (as degree heating weeks from field data DHW_f_) for each site (solid line represents the linear regression model: *r*^2^ = 0.40, *p* = 0.027; the grey area denotes the 95% CI). (Online version in colour.)

*In situ* temperature data show that internal waves coincided with a period of anomalously warm SSTs in the Andaman Sea in May 2010, leading to intermittent periods of cooling near the seabed in shallow (15 m) reef areas (figure 1*b*,*c*; electronic supplementary material, figure S2). The temperature dropped down to a minimum value of 22.1°C measured at the exposed side of Miang during the heat stress period (figure 1*b* and table 1). LAIW cooling intensity differed between exposed sites and was stronger for Miang and Tachai (degree-days cooling of −19.2 and −16.8°C d, respectively) compared with Surin (−10.6°C d, table 1). These differences in cooling and their potential to alleviate heat stress are not reflected in degree heating weeks derived from satellite temperature data (DHW_s_, table 1). By contrast, LAIW resulted in remarkable differences in heat stress when calculated from *in situ* temperature data (DHW_f_, table 1). These data revealed a 40–80% higher heat stress on the sheltered east sides. This is consistent with a significantly reduced BR at LAIW exposed compared with sheltered sites (table 2 and figures 1*d*–*g*, 2*a*; two-tailed *t*-test: *t* = −2.3794, d.f. = 9.97, *p* = 0.039) and suggest that LAIW abate heating and mitigate coral bleaching. Hence, satellite-derived temperature data were not able to predict the observed BR across all study sites (regression slopes not different from 0; table 3). By contrast, DHW_f_ explained 40% of the observed BR (figure 2*b*; *F* = 6.70, *p* = 0.027, d.f. = 10; table 3).

**Table 1.** Temperature conditions during the high temperature anomaly in 2010 summarized for the exposed west (W) and sheltered east (E) sites; Tachai W (TW), Payang W (PW), Miang W (MW), Similan W (SiW), Racha W (RW), Surin W (SuW), Bon W (BW), Racha E (RE), Payang E (PE), Miang E (ME), Similan E (SiE) and Surin E (SuE). Temperature values include mean, maximum (max) and minimum (min) temperature recorded during this period. LAIW cooling intensities are calculated as degree-day cooling (DDC) below the NOAA bleaching threshold (30.62°C). Degree heating weeks (DHW) derived from satellite (DHW_s_) and field data (DHW_f_) reflect differences in heat anomaly observed at the sea surface and in 15 m water depth.

sites	exposed							sheltered				
	TW	PW^a^	MW	SiW^a^	RW	SuW	BW	RE^b^	PE^a^	ME	SiE^a^	SuE
mean (°C)	30.3	—	30.3	—	30.3	30.5	30.6	30.7	—	30.6	—	30.8
max (°C)	32.1	—	31.9	—	31.8	32.2	32.3	32.3	—	32.4	—	32.2
min (°C)	22.1	—	22.1	—	24.5	23.9	23.4	25.7	—	26.3	—	27.6
DDC (°Cd)	−16.8	—	−19.2	—	−15.0	−10.6	−7.7	−10.1	—	−3.9	—	−1.5
DHW_s_	5.2	4	4	4	5.7	6.8	5.2	5.7	4	4	4	6.8
DHW_f_	2.4	2.3	2.3	2.3	1.2	5.4	6.2	6	7.8	7.8	7.8	9

^a^Payang E, Payang W, Similan E and Similan W data are unavailable. For calculation of DHW_f_, temperature data were taken from corresponding E and W sites of Miang, respectively. For justification cf. Schmidt *et al.* [15].

^b^Racha E temperature record was available only for 20 and 10 m water depth. Values for 15 m water depth were obtained by linear interpolation.

**Table 2.** BR and CBSI calculated for all exposed west (W) and sheltered east (E) island sides (abbreviations are same as in table 1) for the bleaching monitoring in May (BR_M_, CBSI_M_) and December 2010 (CBSI_D_, BR_D_).

sites	exposed							sheltered				
	TW	PW	MW	SiW	RW	SuW	BW	RE	PE	ME	SiE	SuE
BR_M_	63.5	47.9	41.8	62.1	39.0	45.2	61.4	52.6	64.6	71.4	70.4	61.2
BR_D_	36.9	20.6	22.7	21.7	27.1	15.6	23.5	21.2	21.9	26.3	20.1	27.2
CBSI_M_	60.4	62.5	59.1	68.7	41.1	36.0	59.5	63.4	69.0	68.2	59.1	53.4
CBSI_D_	51.2	42.9	52.5	51.3	39.8	17.3	50.7	42.6	49.2	44.1	49.0	46.4

**Table 3.** (a–c) Simple and (d–f) multiple linear regression models were calculated with (BR_M_) as response variable and degree heating weeks derived from satellite (DHW_s_) and calculated from *in situ* field data (DHW_f_) as well as CBSI_M_ during heat stress in May 2010 as predictive variables. Significance of *p*-values is denoted by asterisks.

parameter	BR_M_				
	*r*^2^	*F*-statistic	d.f.	*t*-value (slope)	*p*-value
(a) DHW_s_	0.11	1.176	10	−0.86	0.304
(b) DHW_f_	0.40	6.703	10	2.59	0.027*
(c) CBSI_M_	0.39	6.364	10	2.52	0.030*
(d) DHW_s_ + CBSI_M_	0.43	3.382	9		0.080
DHW_s_				0.796	0.447
CBSI_M_				2.259	0.050
(e) DHW_f_ + CBSI_M_	0.67	9.267	9		0.007**
DHW_f_				2.80	0.021*
CBSI_M_				2.74	0.023*
(f) DHW_f_ + CBSI_M_^a^	0.78	14.00	8		0.002**
DHW_f_^a^				3.74	0.006**
CBSI_M_^a^				2.91	0.019*

**p < 0.01, *p < 0.05.

^a^Refit of model (e) by omitting the site Tachai W from the model calculations (see Material and methods section for justification).

Remote sensing has considerably advanced the predictability of coral mass bleaching over recent decades [42], despite challenges and limitations, particularly regarding spatial and temporal resolution [32,43], as well as a general underestimation of temperature values in southeast Asia [44,45]. A notable shortcoming with SST remote sensing is its restriction to the uppermost skin of the ocean surface and, hence, its inability to detect subsurface processes acting on the seabed surrounding the corals. Short-term temperature fluctuations at the study sites and elsewhere are thus not adequately assessed [14,32].

Here we were able for the first time to quantify the strong discrepancy between satellite and *in situ* data during an unprecedented bleaching event. This challenges the applicability of satellite temperature data alone to predict bleaching intensity and patterns in this region and regions with similar variation in temperature regime [14,32]. Even though remotely sensed SST measurements do not capture subsurface LAIW cooling, surface rip bands associated with LAIW can be tracked from space using synthetic aperture radar (SAR) and optical sensors such as MODIS (Moderate Resolution Imaging Spectrometer), allowing the generation of a global LAIW atlas [19]. So far it is not possible, however, to assess the magnitude of LAIW and associated mixing from SAR or MODIS data. The implementation of an *in situ* temperature monitoring system is consequently essential to quantify the magnitude of LAIW-associated cooling. Such information can be used to model the reliability of LAIW reef refugia in a warming ocean [13,46]. Given the ubiquity of LAIW [19], they may rival or exceed the importance of coastal upwelling in mitigating heat stress in corals.

**Figure 3.**
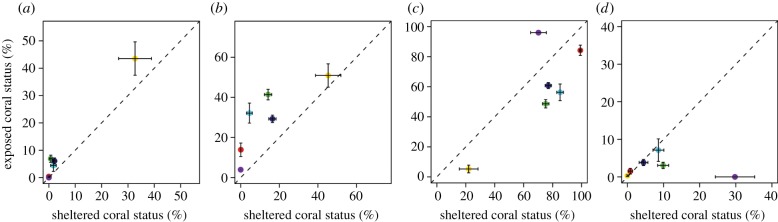
Coral group status during the bleaching event in May 2010, for Pocilloporidae (red), *Acropora* spp. (cyan), *Porites* spp. massive (green), *Porites* spp. branching (purple), other (blue), *Diploastrea heliopora* (yellow). Coral group status recorded as (*a*) healthy, (*b*) pale, (*c*) bleached and (*d*) recently dead and displayed as a fraction of total coral group cover for the sheltered versus exposed island sides during the bleaching event in May 2010.

Owing to its intermittent nature, LAIW cooling is not expected to completely nullify heat stress. Thus, bleached corals were observed at all sites during this severe heat stress and only a small percentage of corals remained healthy two months after the temperature had started to exceed the bleaching threshold. However, a greater percentage of healthy and pale corals were observed at the LAIW-exposed sites while the percentage of bleached and recently dead corals was higher at the LAIW-sheltered sites (figure 3). This was particularly apparent when comparing exposed with sheltered sites of the same island (e.g. BR differences sheltered versus exposed: Racha = 52.6 versus 39.0, Miang = 71.4 versus 41.8 and Surin = 61.2 versus 45.2; table 2).

As coral species differ in their susceptibility to bleaching, coral community composition is a crucial parameter to explain small-scale bleaching variability [34,39]. Therefore, we quantified for each site a CBSI to rank sites according to their intrinsic bleaching susceptibility. Bleaching vulnerable coral communities were found on both exposed and sheltered sites (e.g. exposed side of Similan and the sheltered sides of Payang and Miang; table 2). However, some susceptible groups like branching *Porites* were almost exclusively observed at the sheltered island sides (see the electronic supplementary material, figure S3a). Individual coral groups showed strong differences in the extent of bleaching in May 2010 with milder bleaching on the exposed sites even within the most susceptible group (figure 3). Community inherent differences in bleaching susceptibility alone explained 39% of the observed BR (*F* = 6.364, *p* = 0.030, d.f. = 10; table 3) and already provide an estimation of how severe a BR will be for different reef communities. However, it is still unknown whether susceptibility patterns undergo substantial changes after this bleaching event, which has not been observed so far on the Thai coast of the Andaman Sea [34], but has been shown for other locations in southeast Asia [47].

Extrinsic (environmental conditions) and intrinsic factors (community assemblage) are quite well able to predict differences in bleaching severity. Incorporating both extrinsic and intrinsic factors in models proved essential for increasing bleaching prediction accuracy [48,49]. The predictability of our models increased markedly, explaining 67% of the BR by taking both DHW_f_ and CBSI_M_ into account (*F* = 9.267, *p* = 0.007, d.f. = 9; table 3). Our combined linear regression model provides some key messages: first of all it highlights the potential of LAIW to provide protection from mass bleaching, and second it underlines the need to monitor temperature as well as community composition at the reef scale.

Variability in BR has been further attributed to other extrinsic (e.g. turbulence, light) and intrinsic factors (e.g. energy reserve, thermal history). At the study sites, both LAIW and the SW monsoon increase currents, which are known to reduce the BR [50–52]. Previous thermal history, in particular exposure to substantial temperature fluctuations, can render corals more stress-resistant [53–55]. While most of these studies investigated warm temperature anomalies, the negative anomalies observed here on the exposed sites might have a similar effect on the BR. Variations in content, composition and acquisition of energy reserves can allow corals to better cope with heat stress [56,57]. LAIW exposure affects the energetic status of corals [58,59] by the delivery of plankton and nutrients into the reef [15,58]. This additional energy supply may also account for a reduced BR. Bleaching intensity is also a matter of exposure to the intensity of solar radiation with increased light levels to cause bleaching [60]. Both the arrival of LAIW and the impact of the SW monsoon waves increase turbidity and sedimentation in shallow water areas [61], resulting in reduced light levels at the western LAIW-exposed sites. However, increased sedimentation can also be stressful to corals [62,63]. Hence, the corals themselves might be more robust against thermal stress and may benefit from increased water currents in addition to LAIW cooling, but may be negatively affected by the increased SW monsoon sedimentation rate.

Surveys carried out half a year after the bleaching monitoring (December 2010) revealed that the surviving corals had started to recover, but were not fully recovered yet (BR is not 0; table 2; corals still showed signs of bleaching; electronic supplementary material, figure S1b1–2). Despite the strong LAIW-related differences in bleaching mitigation during the thermal stress period, recovery of the remnant coral community was not very different between exposed and sheltered sites (two-tailed *t*-test BR exposed versus sheltered sites: *t* = 0.2332, d.f. = 9.081, *p* = 0.82). This suggests that LAIW and monsoon exposure on the W sides of the islands may play an antagonistic role (i.e. mitigating bleaching during the dry season but delaying recovery during the SW monsoon). The SW monsoon is characterized by increased sedimentation rates on the exposed sites at the height of the wet season [16]. Sediment removal is energetically expensive for the corals, potentially diverting a higher fraction of the available energy away from regeneration and repair. Coral photosynthesis is also reduced in turbid waters [62,63]. Both the reduced energy from photosynthesis and the reallocation of energy to remove the sediment is likely to have hampered the recovery process on the exposed reefs.

**Figure 4.**
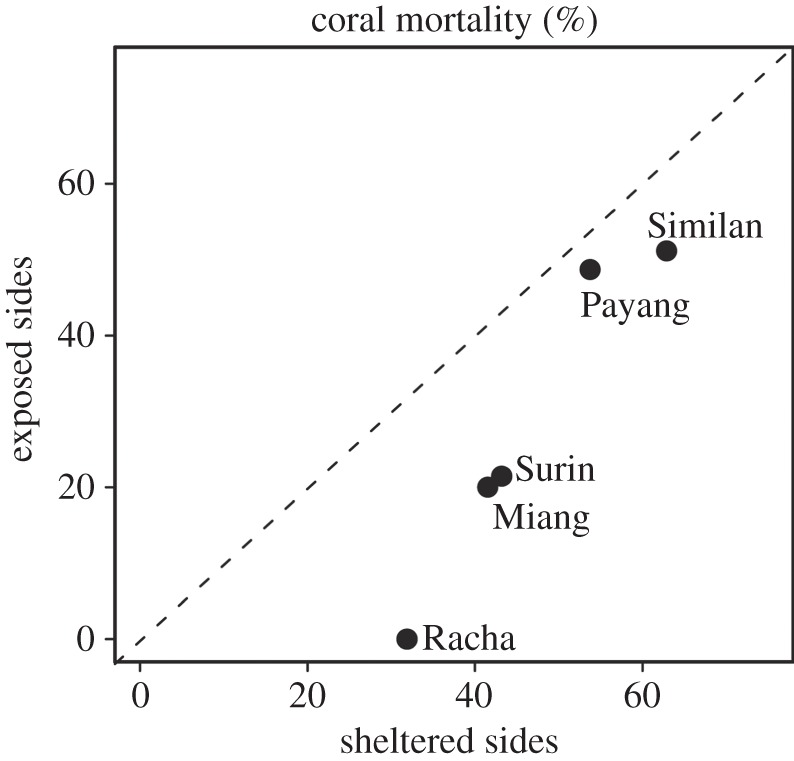
Scatter plot of coral mortality observed in December 2010 on sheltered compared with the respective exposed site.

By contrast, mortality at exposed and sheltered sites showed strong differences, with higher post-bleaching mortality on sheltered compared with the respective exposed site (figure 4). In addition, mortality varied strongly between species, resulting in post-bleaching coral communities with a higher proportion of resilient taxa at all sites (table 2; CBSI_D_ during recovery phase: 44.8 ± 3.0 versus CBSI_M_ during the thermal stress period: 58.4 ± 2.8). This caused a shift in the dominance of coral taxa across sites, resulting in coral communities that are more bleaching-resistant (i.e. that have a lower CBSI). Whether such a shift represents an alternative state or a long-lasting condition strongly depends on the frequency and intensity of bleaching events.

The coral community at Tachai W showed severe bleaching and the slowest recovery (table 2) despite both strong LAIW cooling (table 1; see the electronic supplementary material, figure S2) and moderate sedimentation rates [16]. This discrepancy can only partly be explained by the high percentage of bleaching-susceptible species found at this site (table 2). High densities of corallimorpharians were observed at Tachai W accounting for 42% of the benthic cover (see the electronic supplementary material, table S1). Corallimorpharians have been described as aggressive space competitors, which may kill corals at early stages of succession following disturbance events [64]. Corallimorpharians were already present at this site during the thermal anomaly and were observed to compete for space with *Porites lutea* (see the electronic supplementary material, figure S4). This may have exacerbated the physical stress conditions (high temperature during the thermal anomaly and sedimentation during the SW monsoon), which highlights the complexity of factors and interactions governing the reef ecological responses to a changing environment. Multiple stressors are well known to additionally reshape coral reef communities [65,66], and therefore need careful consideration when monitoring reef condition and predicting future reef trajectories [67,68].

It has been predicted that bleaching events will occur annually or biannually by 2050 [7], with critical consequences for reef health and distribution [2]. The observed community shift towards more heat-tolerant species composition potentially renders coral communities more resistant to the predicted future bleaching scenarios [69]. However, this may occur at the expense of species diversity [65]. Bleaching-susceptible species survived at the exposed sites (see the electronic supplementary material, figure S3b) and may survive under the predicted future scenario in such natural resilient areas. This enhances their chance to recolonize the sheltered E sites and potentially can help to maintain biodiversity and reef integrity. Both the selection of heat-resistant species on the sheltered sites and the maintenance of coral biodiversity on the exposed sites may prove essential for sustaining coral reefs in the Andaman Sea and other semi-enclosed tropical basins in the face of climate change.

LAIW benefit corals during unprecedented bleaching and LAIW-exposed coasts may provide local refugia for corals. Because LAIW are ubiquitous in tropical areas, they may play a major role in sustaining coral diversity and cover in a warming climate. LAIW exposure is, however, a ‘mixed blessing’, as it hampers reef development [15,16] and coral growth [70], but promotes high diversity [15] and coral fitness [58,59] under prevailing conditions (i.e. non-bleaching) that might prove essential for reef persistence in this area. While LAIW have proved beneficial in alleviating thermal stress, sedimentation caused by increasing monsoon swell appears to retard the recovery process. While both processes are spatially coupled (both act from a westerly direction), they are temporally decoupled. In other non-monsoonal settings, the situation may be simpler and coral recovery will probably be faster. Internal wave-induced temperature variations were observed in tropical reefs to range from 1–3°C [20,21,26,27] up to 10°C [14,24,28,32], and differ in frequency and duration. In the Caribbean, for instance, internal tides yield cold-water periods that are not as sudden and short-lived [20,32] as in the Andaman Sea. Consequently, the effects on coral growth, reduced reef development [20] and potentially species diversity appear much less pronounced. However, additional stressors that lead to a dramatic decline in coral health [71] might represent the bottleneck for coral resistance to future changes. Our study highlights how a complex suite of environmental and biological factors interact to explain coral bleaching and recovery at the local scale. Understanding the physical dynamics and ecological responses is instrumental to understand the resilience of corals in a changing climate. LAIW may play an important, yet understudied role in providing local refugia for corals in a warming world.

## Supplementary Material

Wall_eta_Proceedings_SI

## Data accessibility

Data are available through the Pangaea data repository.
