# Supplementary material for: Large-amplitude internal waves benefit corals during thermal stress
Source: Proc Biol Sci. 2015 Jan 22;282(1799):20140650. doi: 10.1098/rspb.2014.0650 (PMC4286055; doi:10.1098/rspb.2014.0650)
Supplement: Wall_eta_Proceedings_SI [file rspb20140650supp1.docx]

Electronic Supplementary Material to:

**LARGE AMPLITUDE INTERNAL WAVES BENEFIT CORALS DURING THERMAL STRESS**

**M Wall^1,2,*^, L Putchim^1,3^, GM Schmidt^1^, C Jantzen^1^, S Khokiattiwong^3^ & C Richter^1^**

**^1^***Alfred Wegener Institute, Helmholtz Center for Polar and Marine Research,*

*Am Alten Hafen 26, 27568 Bremerhaven, Germany*

**^2^***GEOMAR, Helmholtz Center for Ocean Research, Marine Geosystems,*

*Wischhofstraße 1-3, 24148 Kiel, Germany*

**^3^***Phuket Marine Biological Center,*

*51 Sakdidet Road, 83000 Phuket, Thailand*

**Sublementary Matrials and Methods:**

1. (d) Data analysis:

(i) Temperature analysis – site specific temperature indices:

Satellite degree heating weeks (DHW_s_) were derived from the ReefGIS data base (http://reefgis.reefbase.org/ default.aspx?wms=RGWDHW provided by ReefBase) by using the maximum DHW value determined for May 2010 (the time of the thermal stress period and bleaching monitoring) for each location. Where data were unavailable (e.g. Tachai and Bon were located too close to land with no valid SST estimate), the nearest pixels were used.

In situ temperature data were decomposed into weekly means and residuals to the long-term climatological maximum monthly mean SST (MMM) to calculate temperature anomalies as DHW_f_ [1]. DHW_f_ were derived for the time of bleaching monitoring in May 2010 by considering the weakly residuals of the 12 weeks prior to that time point; residuals were only summed if they were ≥ 1°C (i.e. temperature above the NOAA bleaching threshold of 30.62°C).

To calculate MMM long-term temperature data were derived from remotely sensed SST using Advanced Very High Resolution Radiometer (http://ncdc.noaa.gov/thredds/dodsC/oisst/NetCDF/AVHRR/, OISST-V2-AVHRR_agg, Long: 97.4E, Lat: 8.4N) for the time period 1985 to 2009 (MMM = 29.62°C).

In order to receive a measure of the intensity of LAIW cooling the temperature anomalies were calculated as cumulative degree days (DDC in [°C d]) according to Leichter and Genovese [2]. Here we only considered temperature drops as cooling during the heat anomaly period if they fell below the NOAA bleaching threshold (30.62°C). Each single temperature value was subtracted from the bleaching threshold. The negative temperature anomalies were integrated over time.

**Supplementary tables:**

**Table S1.** Cover of corallimorpharian at each site during thermal stress and recovery period. Percentage of corallimorpharian cover for the different exposed west (W) and sheltered east (E) sites (Tachai W (TW), Payang W (PW), Miang W (MW), Similan W (SiW), Racha W (RW), Surin W (SuW), Bon W (BW), Racha E (RE), Payang E (PE), Miang E (ME), Similan E (SiE) and Surin E (SuE)) observed during the bleaching monitoring in May and recovery period in December 2010 in 15 m water depth.

|  | **% cover corallimorpharian** | |
| --- | --- | --- |
| **sites/time** | **May 2010** | **December 2010** |
| TW | 41.85 | 33.81 |
| PW | 0.00 | 0.00 |
| MW | 0.00 | 0.00 |
| SiW | 0.00 | 0.00 |
| RW | 0.92 | 0.00 |
| SuW | 0.00 | 0.00 |
| BW | 0.00 | 0.00 |
| RE | 0.00 | 0.00 |
| PE | 0.00 | 0.00 |
| ME | 0.00 | 0.00 |
| SiE | 0.00 | 0.00 |
| SuE | 0.00 | 0.00 |

**Supplemental figures:**

**
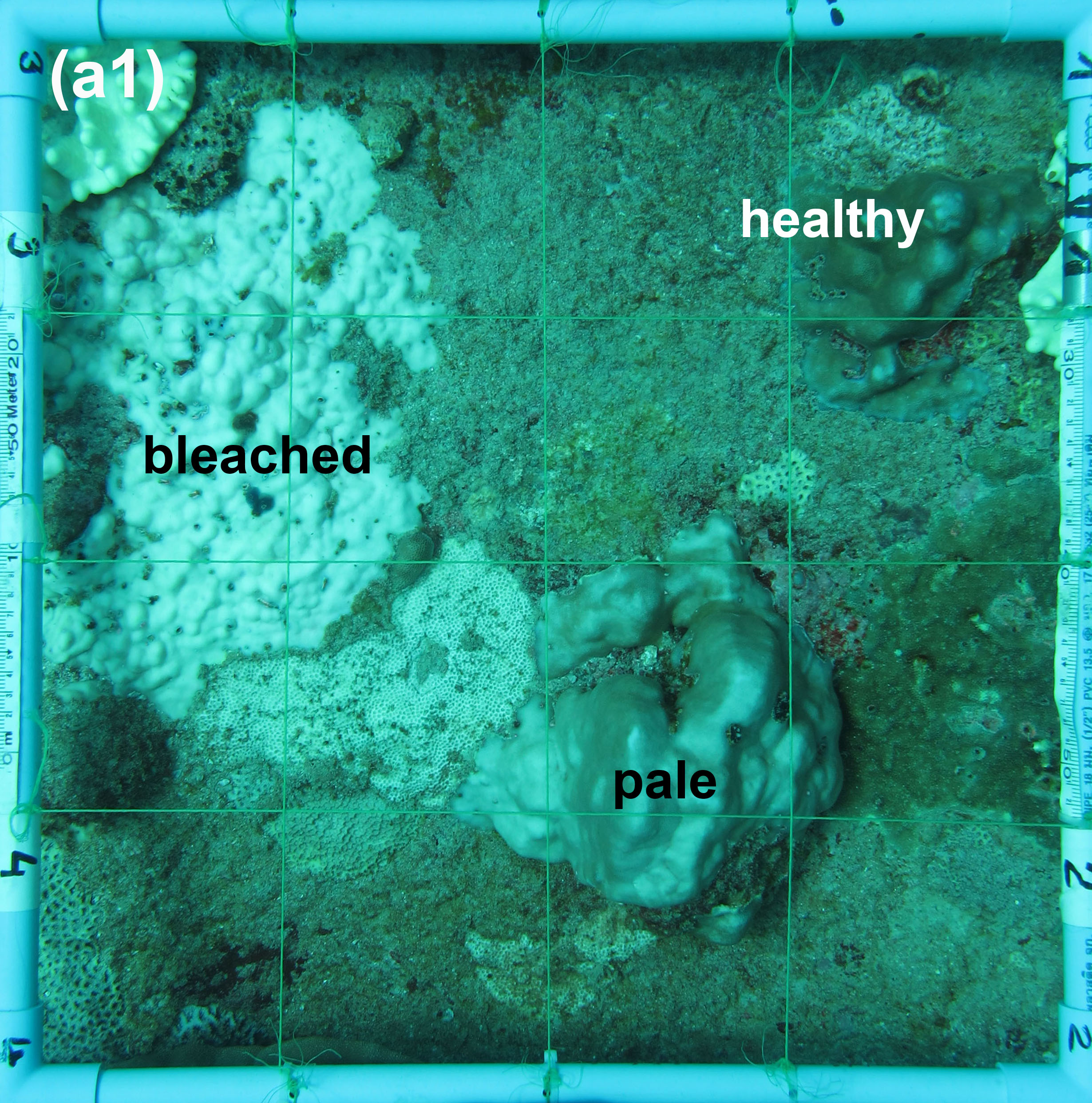

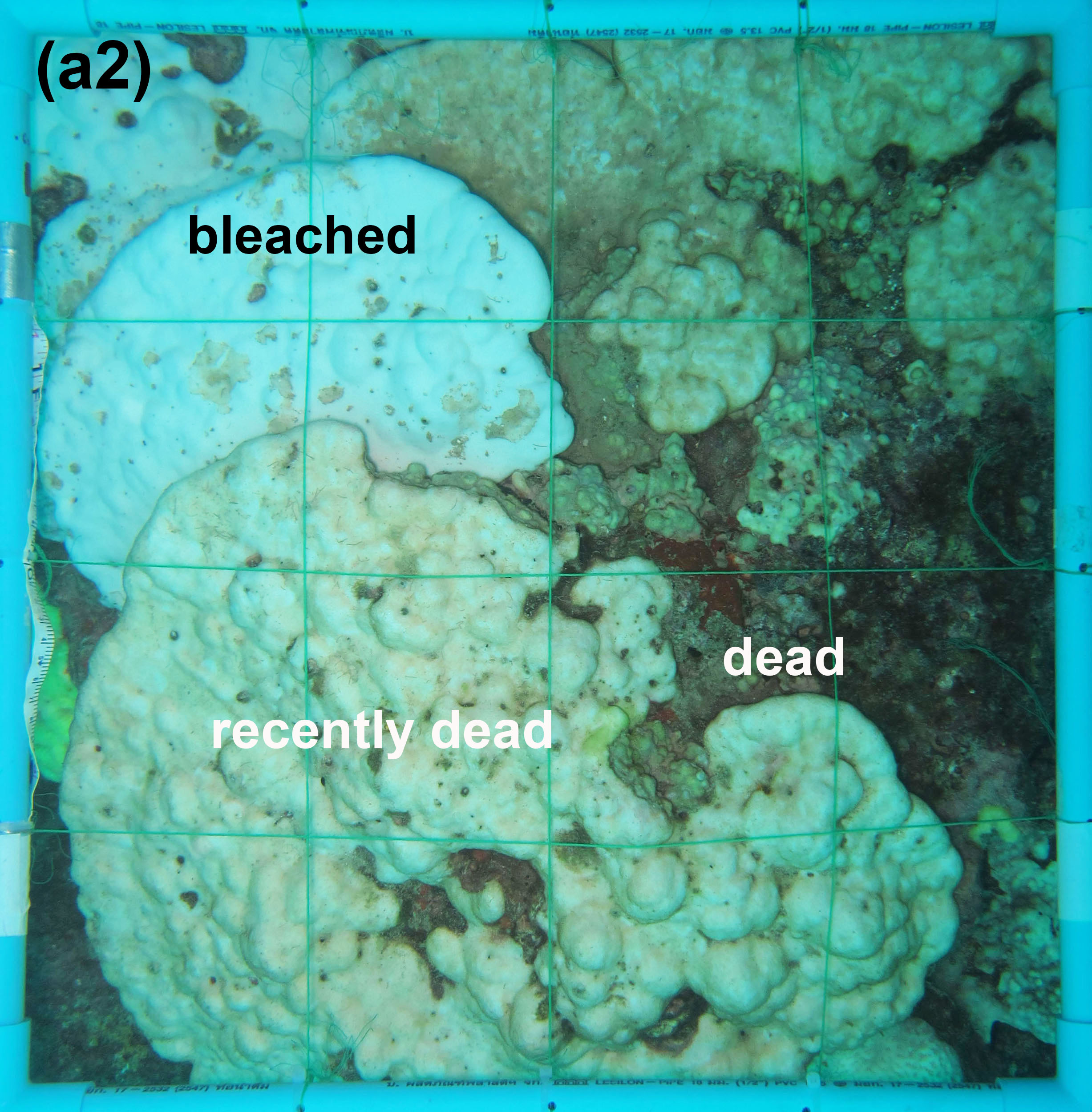
**

**
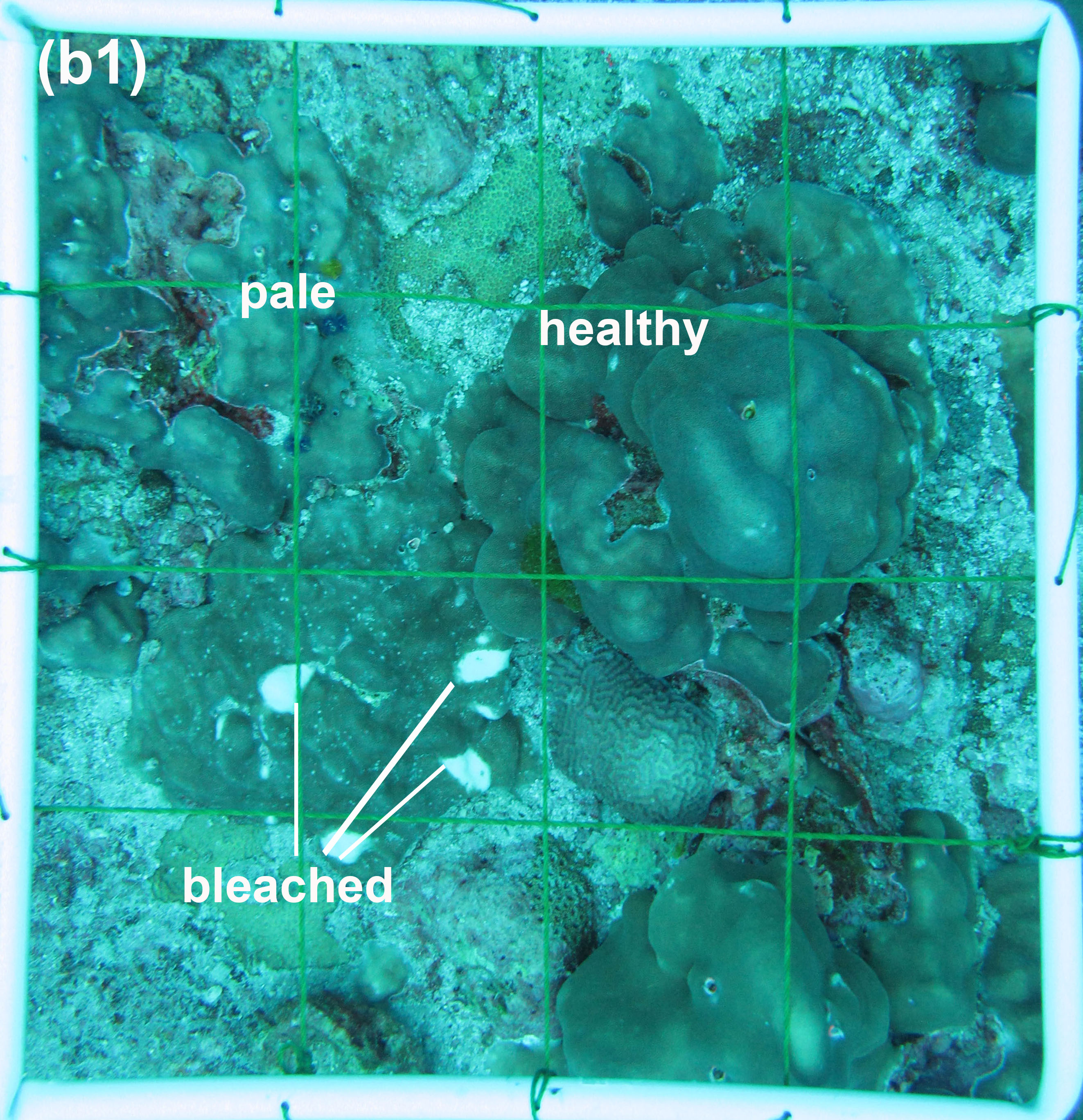

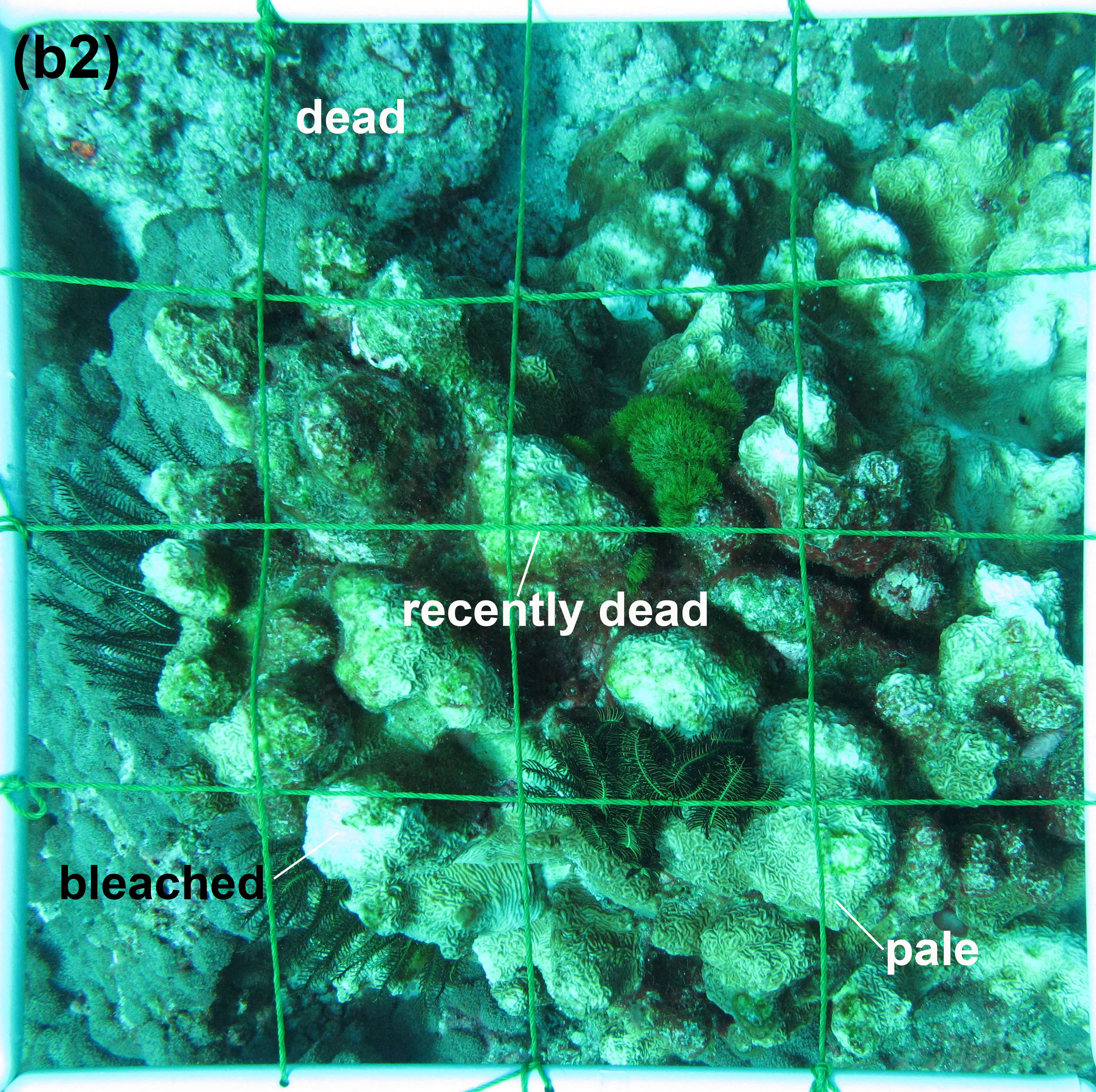
**

**Figure S1.** Photoquadrats taken in May (a1,2) and December (b1,2) that illustrate the different bleaching categories (“healthy”, “pale”, “bleached”, “recently dead” and “dead”).

**
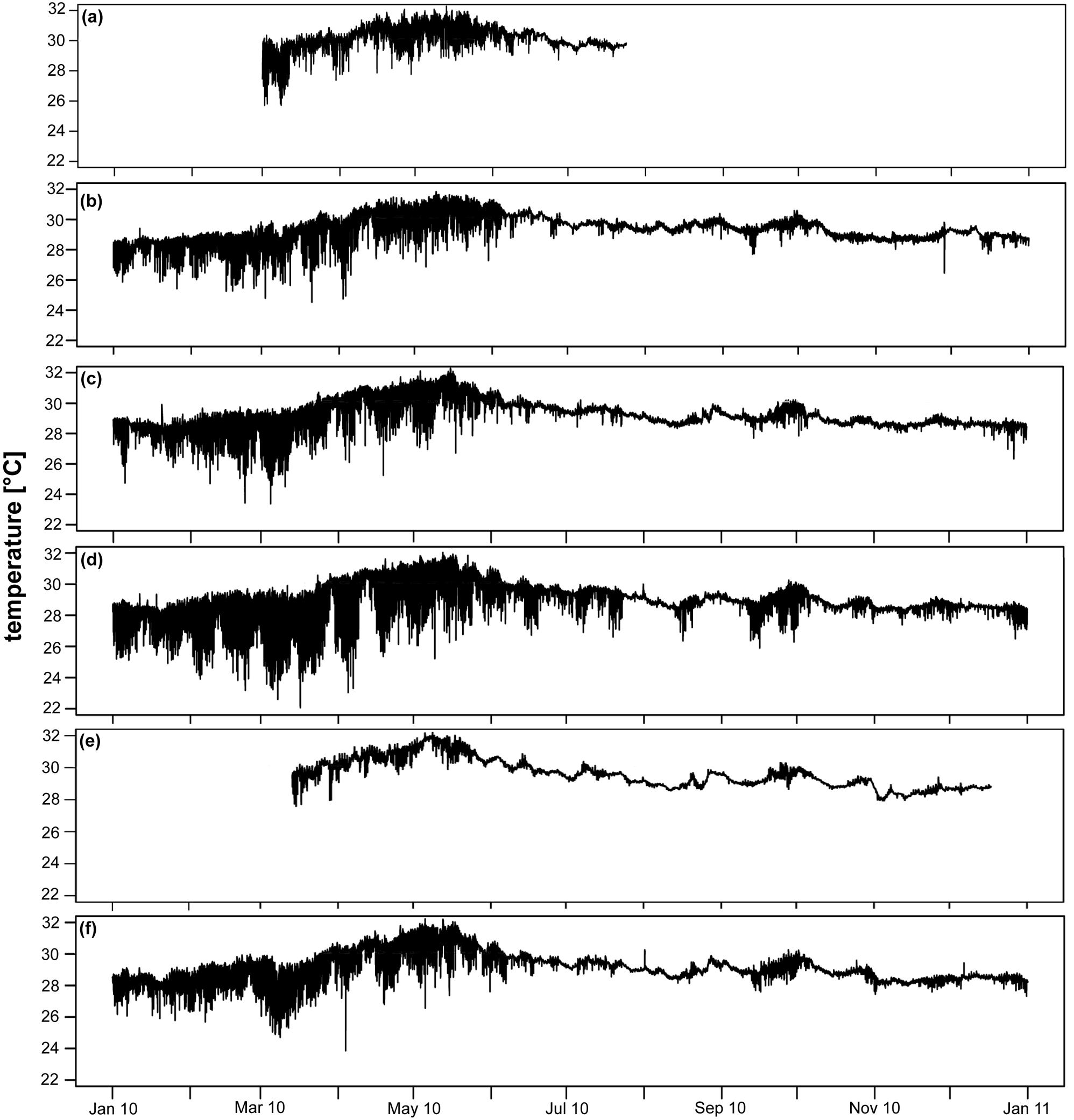
**

**Figure S2.** Temperature record from additional sites for the period logger data were available. Temperature record during the bleaching year 2010 derived for exposed west (W) and sheltered east (E) sites ((a) Racha E, (b) Racha W, (c) Bon W, (d) Tachai W, (e) Surin E and (f) Surin W) measured at 15 m water depth expect Racha East temperature record derived from 20 m water depth.


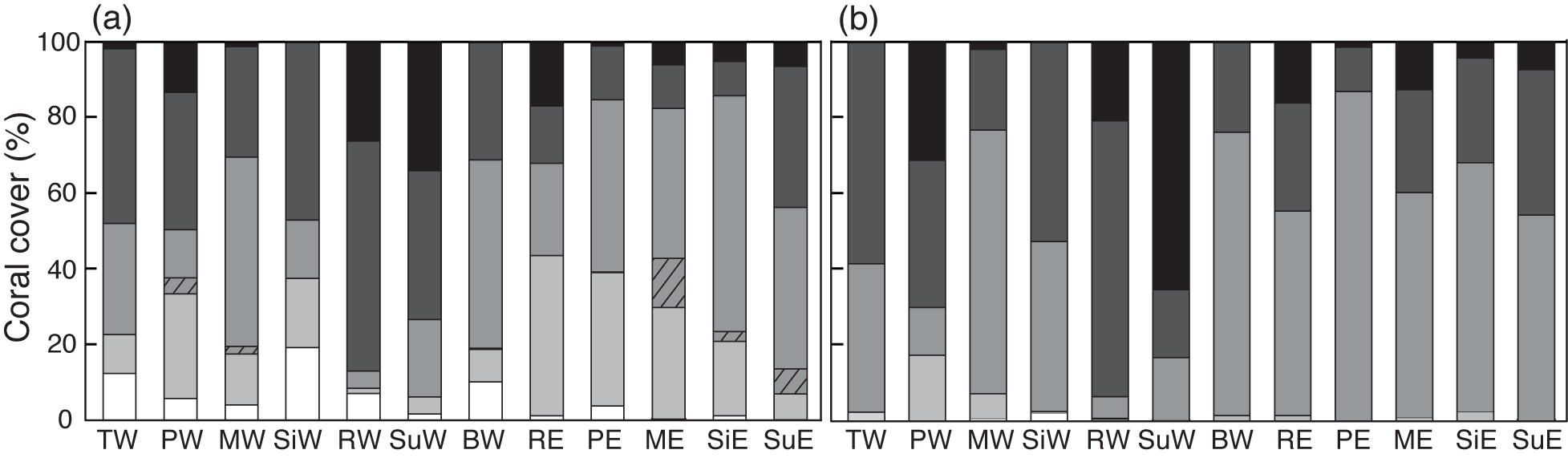


**Figure S3.** Coral community composition. Coral community composition (Pocilloporidae (white), *Acropora* (light grey), *Porites* branching (grey-stripped), *Porites* massive (grey), other (dark grey) and *Diploastrea* (black) arranged from bottom to the top from the most bleaching susceptible to least susceptible taxa) as a fraction of coral cover during the bleaching event in May (a) and recovery phase in December 2010 (b). All data are displayed for all exposed west (W) and sheltered east (E) sites (Tachai W (TW), Payang W (PW), Miang W (MW), Similan W (SiW), Racha W (RW), Surin W (SuW), Bon W (BW), Racha E (RE), Payang E (PE), Miang E (ME), Similan E (SiE) and Surin E (SuE)). Sites are arranged from highest to lowest cooling left to right.


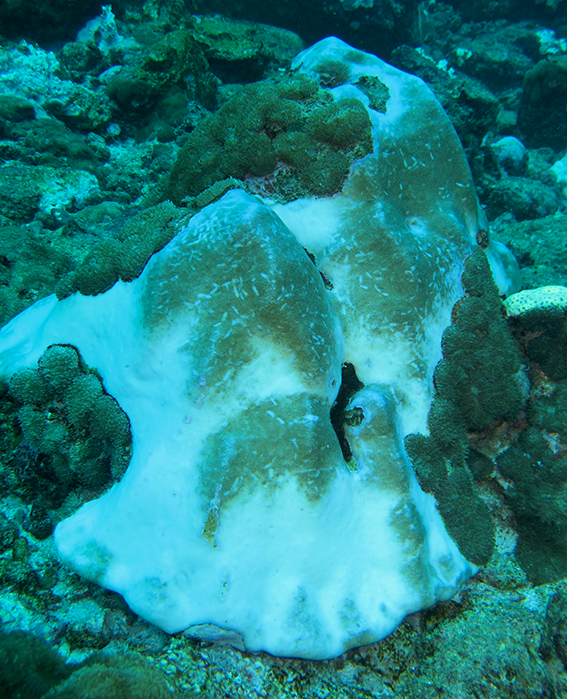


**Figure S4.** Corallimorpharian space competitors. Corallimorpharian space invaders impact the reef in Tachai west covering 42% of the available substrate. Image was taken during the bleaching monitoring in May 2010.

**References:**

1. Liu, G., Strong, A. E. & Skirving, W. J. 2003 Remote sensing of sea surface temperature during 2002 Barrier Reef coral bleaching. *EOS* **84**, 137-144.

2. Leichter, J. J. & Genovese, S. J. 2006 Intermittent upwelling and subsidized growth of the scleractinian coral *Madracis mirabilis* on the deep fore-reef slope of Discovery Bay, Jamaica. *Mar. Ecol. Prog. Ser.* **316**, 95-103.
